# Poisoning by Cyder Containing Lead in Solution

**Published:** 1842-11-19

**Authors:** 


*Poisoning by Cyder containing Lead in Solution.*—Six individuals, after drinking cyder for a few days, were seized with the usual symptoms of poisoning by lead, as violent colic, obstinate constipation, pains in limbs, with the attendant trembling. They recovered under the usual remedies. MM.

Chevallier, Olivier, and Pages were directed to investigate into the mode in which the cyder had become adulterated with lead, and found, that, during its preparation, it had been allowed to remain for two days in a reservoir which was lined with lead. They also recognized that the salt of lead which existed in the cyder was a malate, which they found to be a soluble salt. The only interest of this case depends on this circumstance, as both Thomson and Berzelius describe the malate of lead as an insoluble salt; and it is well that chemists should be aware of the fact of its solubility.—*Ibid, from Annales d'Hygiene Publique*, January, 1842.

